# Treatment with atrial natriuretic peptide induces adipose tissue browning and exerts thermogenic actions in vivo

**DOI:** 10.1038/s41598-021-96970-9

**Published:** 2021-08-31

**Authors:** Haruka Kimura, Tomohisa Nagoshi, Yuhei Oi, Akira Yoshii, Yoshiro Tanaka, Hirotake Takahashi, Yusuke Kashiwagi, Toshikazu D. Tanaka, Michihiro Yoshimura

**Affiliations:** Division of Cardiology, Department of Internal Medicine, The Jikei University School of Medicine, 3-25-8, Nishi-Shimbashi, Minato-ku, Tokyo, 105-8461 Japan

**Keywords:** Cardiology, Cardiovascular biology, Cardiovascular diseases, Heart failure

## Abstract

Increasing evidence suggests natriuretic peptides (NPs) coordinate inter-organ metabolic crosstalk with adipose tissues and play a critical role in energy metabolism. We recently reported A-type NP (ANP) raises intracellular temperature in cultured adipocytes in a low-temperature-sensitive manner. We herein investigated whether exogenous ANP-treatment exerts a significant impact on adipose tissues in vivo. Mice fed a high-fat-diet (HFD) or normal-fat-diet (NFD) for 13 weeks were treated with or without ANP infusion subcutaneously for another 3 weeks. ANP-treatment significantly ameliorated HFD-induced insulin resistance. HFD increased brown adipose tissue (BAT) cell size with the accumulation of lipid droplets (whitening), which was suppressed by ANP-treatment (re-browning). Furthermore, HFD induced enlarged lipid droplets in inguinal white adipose tissue (iWAT), crown-like structures in epididymal WAT, and hepatic steatosis, all of which were substantially attenuated by ANP-treatment. Likewise, ANP-treatment markedly increased UCP1 expression, a specific marker of BAT, in iWAT (browning). ANP also further increased UCP1 expression in BAT with NFD. Accordingly, cold tolerance test demonstrated ANP-treated mice were tolerant to cold exposure. In summary, exogenous ANP administration ameliorates HFD-induced insulin resistance by attenuating hepatic steatosis and by inducing adipose tissue browning (activation of the adipose tissue thermogenic program), leading to in vivo thermogenesis during cold exposure.

## Introduction

A-type natriuretic peptide (ANP) as well as B-type natriuretic peptide (BNP), which are hormones produced in the heart, regulate blood pressure and fluid homeostasis through vasodilatory and diuretic actions, and improve cardiac remodeling^1–4^. In addition to these classical actions of hemodynamic regulation on the renal and cardiovascular systems, growing evidence suggests that natriuretic peptides (NPs) also regulate the energy balance and glucose homeostasis as well as thermogenesis through interorgan metabolic crosstalk with adipose tissues, in which NP receptors (NPR-A) are expressed^4–12^.

Adipose tissues are broadly classified into two general categories: white adipose tissue (WAT) and brown adipose tissue (BAT). WAT primarily functions as an energy storage depot and a source of adipokines, which induce insulin resistance and are responsible for metabolic disorders. In contrast, BAT promotes energy utilization, leading to the reduction of insulin resistance, and is also considered the major site of non-shivering thermogenesis and heat generation using metabolic fuel^7,9,13,14^. Uncoupling protein 1 (UCP1) is specifically expressed in BAT and enables mitochondrial uncoupled respiration, rather than ATP production, allowing for the dissipation of nutritional energy as heat^4,15,16^.

Recently, several studies showed that ANP/BNP promote triglyceride lipolysis as well as uncoupling of mitochondrial respiration by inducing adipose tissue browning, which results in ameliorating insulin resistance and also activating the thermogenic program^4–7,13,14,17–21^. Our recent in vitro study demonstrated that in cultured brown adipocytes, ANP raises the intracellular temperature in a low-temperature-sensitive manner via the activation of the p38-UCP1 pathway^9^. In addition, our clinical study using the cardiac catheter database showed that a decrease in left ventricular ejection fraction is associated with a body temperature decrease, whereas high plasma BNP level is associated with a body temperature increase^4^. These data indicate that NPs have the adaptive heat-retaining property when body temperature falls owing to unfavorable hemodynamic conditions^4,5,9^. On the other hand, both experimental^6,7^ and clinical^10^ studies recently reported that cold exposure per se induces the elevations in ANP/BNP levels, which consistently supports the idea that NPs induce the activation of the adipose tissue thermogenic program in response to cold stimuli as a compensatory mechanism^5,14^. A series of these studies indicated that NPs play a central role in “myocardial-adipose crosstalk”.

To better understand the direct impact of NPs on adipose tissues in vivo and to determine their functional significance, we investigated whether or not exogenous ANP treatment induces WAT “browning”, and promotes BAT activation, as well as whether it attenuates hepatic steatosis, in diet-induced obese mice, resulting in the amelioration of systemic insulin resistance. We also explored the possibility that ANP treatment might exert an adaptive heat-retaining effect during cold exposure through the activation of the adipose tissue thermogenic program in vivo.

## Results

### Effects of ANP treatment on body weight and blood pressure in HFD mice

**Figure 1:**
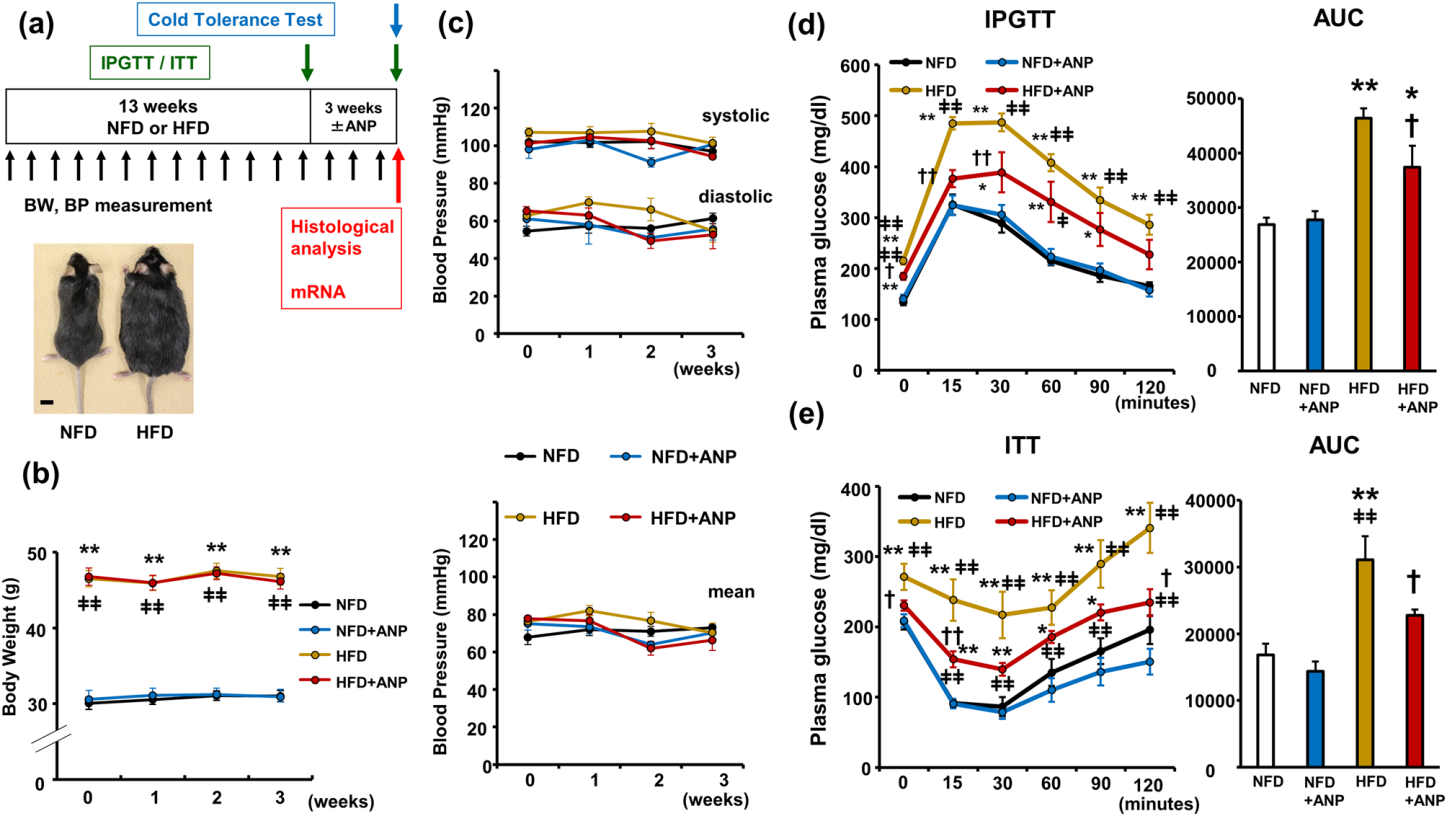
Effects of ANP treatment on HFD-induced obesity and insulin resistance. (**a**) A schematic diagram of the experimental protocol and appearance of the obese mice after 13 weeks of HFD feeding. Bars = 1 cm (**b**) body weight changes during ANP treatment (n = 15 each). (**c**) Blood pressure changes during ANP treatment (n = 9 each). Plasma glucose levels during IPGTT (**d**) and ITT (**e**) at three weeks after treatment with or without ANP ((d) NFD and HFD, n = 8 each; NFD + ANP, n = 9; HFD + ANP, n = 7. (e) NFD, NFD + ANP and HFD, n = 9; HFD + ANP, n = 8.). The area under the curve (AUC) was calculated from the plasma glucose levels profile shown in each test. Data are mean ± SEM. *P < 0.05 and **P < 0.01 versus NFD at each time point; ^†^*P* < 0.05 and ^††^*P* < 0.01 vs. HFD at each time point; ^ǂ^*P* < 0.05 and ^ǂǂ^*P* < 0.01 versus NFD + ANP at each time point. ANP, A-type natriuretic peptide; BP, blood pressure; BW, body weight; HFD, high-fat diet; IPGTT, intraperitoneal glucose tolerance test; ITT, insulin tolerance test; NFD, normal-fat diet.

The study design is shown in Fig. 1a. After 13 weeks of high-fat diet (HFD) feeding, mice developed marked obesity with a significant increase in body weight compared with normal-fat diet (NFD) mice (47.3 ± 0.5 g vs. 31.9 ± 0.9 g, *p* < 0.01, n = 6 each, Fig. 1a). Accordingly, fasting plasma glucose levels were higher in HFD mice than in NFD mice (190.6 ± 11.1 mg/dl vs. 107.5 ± 6.7 mg/dl, *p* < 0.01, Supplementary Fig. S1a). The glucose tolerance test (IPGTT) (Supplementary Fig. S1a) and insulin tolerance test (ITT) (Supplementary Fig. S1b) clearly demonstrated that 13-week HFD feeding induced glucose intolerance and insulin resistance. We confirmed that the ANP administration significantly increased the serum ANP concentration in both NFD (65.4 ± 3.4 pg/ml vs. 176.2 ± 23.6 pg/ml, *p* < 0.05) and HFD mice (155.8 ± 10.0 pg/ml vs. 236.6 ± 26.1 pg/ml, *p* < 0.05) (Supplementary Fig. S4a). ANP treatment for three weeks did not significantly affect the body weight change in either NFD or HFD mice (Fig. 1b). Likewise, blood pressure was not changed by either HFD feeding or treatment with ANP during the experimental protocols in the current model (Fig. 1c).

### ANP treatment improves HFD-induced glucose intolerance and insulin resistance in HFD mice

We next examined the effects of ANP treatment on glucose tolerance and insulin sensitivity. The fasting plasma glucose levels at the beginning of IPGTT in HFD mice was significantly decreased after 3-week ANP treatment (215.1 ± 7.4 mg/dl in HFD vs. 184.4 ± 5.4 mg/dl in HFD + ANP group, *p* < 0.05, Fig. 1d), although the serum insulin concentrations were all below measurable limits at baseline. Furthermore, IPGTT and ITT demonstrated that HFD feeding induced glucose intolerance and insulin resistance, which were ameliorated by ANP treatment (Fig. 1d, e, and Supplementary Fig. S1c,d). These results indicate that ANP treatment significantly improves glucose tolerance and insulin sensitivity in HFD-induced obese mice.

### ANP treatment improves HFD-induced adipose tissue morphological changes and hepatic steatosis

**Figure 2:**
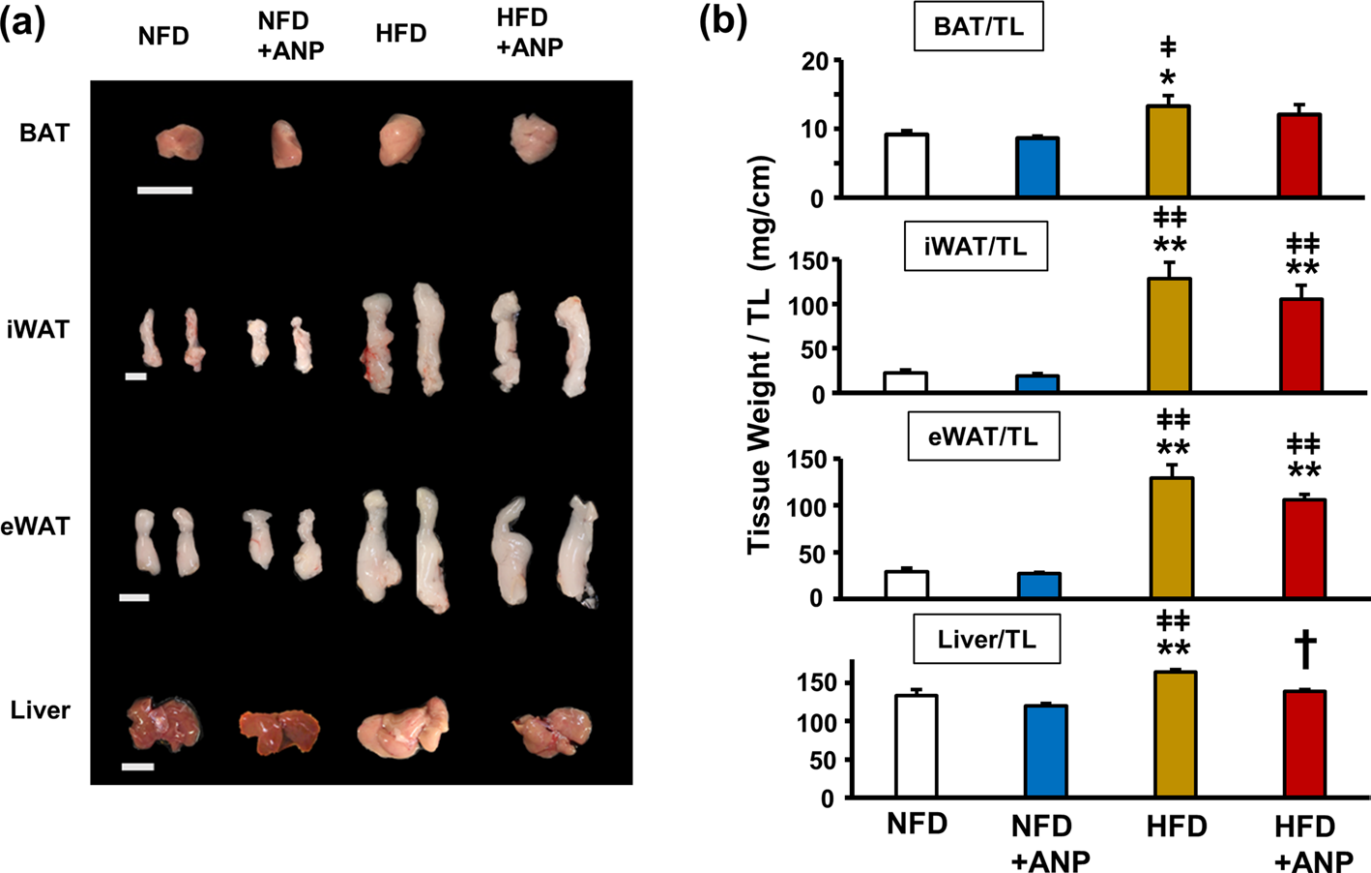
Macroscopic appearances and weights of the adipose tissues and the liver. The representative images (**a**) and weight (**b**) of each tissue harvested from the indicated mice at three weeks after treatment with or without ANP (n = 6 each). Bars = 1 cm. Data are mean ± SEM. *P < 0.05 and **P < 0.01 versus NFD; ^†^*P* < 0.05 versus HFD; ^ǂ^*P* < 0.05 and ^ǂǂ^*P* < 0.01 versus NFD + ANP. BAT, brown adipose tissue; iWAT, inguinal white adipose tissue; eWAT, epididymal white adipose tissue.

**Figure 3:**
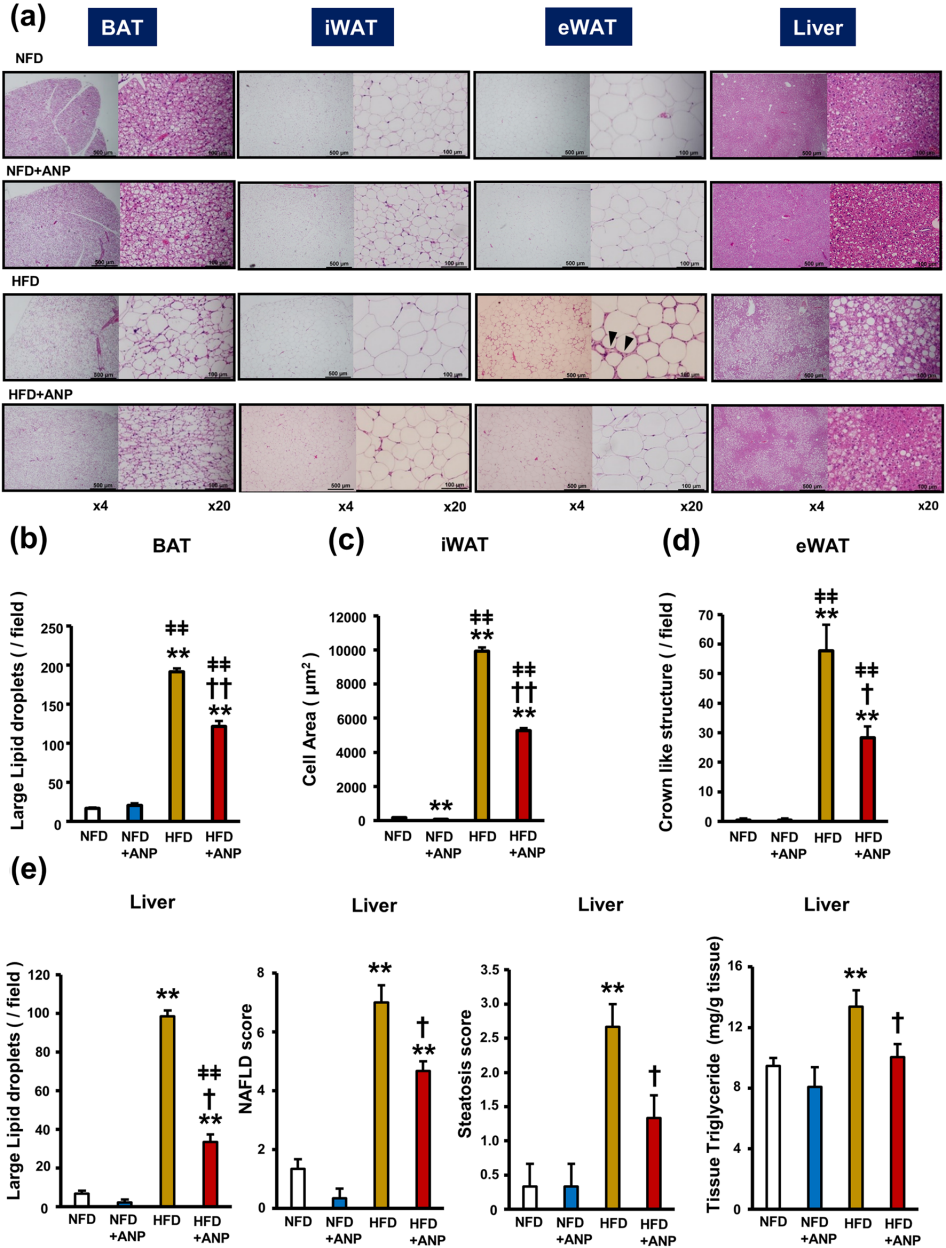
Effects of ANP treatment on HFD-induced enlargement of lipid droplets and inflammation in adipose tissues and hepatic steatosis. (**a**) Representative histological images (hematoxylin–eosin staining) of each tissue harvested from the indicated mice at three weeks after treatment with or without ANP (n = 3 each). The images were captured at a field lens magnification of × 4 (left, bars = 500 μm) and × 20 (right, bars = 100 μm). The arrow heads indicate crown-like structures. (**b**) The number of large lipid droplets (> 100µm^2^)/field in BAT (field lens × 20, n = 3 each). (**c**) The cell area/field in iWAT (field lens × 20, n = 3 each). (**d**) The number of crown like structures/field in eWAT (field lens × 4, n = 3 each). (**e**) The number of large lipid droplets (> 100µm^2^)/field in liver (field lens × 20, n = 3 each). NAFLD score and steatosis score calculated in each group (n = 3 each). Triglyceride content in the hepatic tissues (n = 5 each). Data are mean ± SEM. *P < 0.05 and **P < 0.01 versus NFD; ^†^*P* < 0.05 and ^††^*P* < 0.01 versus HFD; ^ǂ^*P* < 0.05 and ^ǂǂ^*P* < 0.01 versus NFD + ANP.

To clarify the mechanism by which ANP treatment ameliorates insulin resistance in HFD mice, we investigated the influence of ANP on the morphological changes in various adipose tissues and the liver. The weight of all adipose tissues examined as well as the liver were increased in HFD mice compared with NFD mice (Fig. 2a,b). Although ANP treatment did not affect the weight of any adipose tissues, it significantly attenuated HFD-induced hepatomegaly. Histological analyses revealed that HFD feeding resulted in so-called BAT “*whitening*”, which was characterized by increased cell size associated with the accumulation of lipid droplets (Fig. 3a,b). ANP treatment markedly attenuated these histological changes (*re-browning*). Furthermore, HFD feeding induced enlarged lipid droplets of inguinal WAT (iWAT) that were reduced by ANP treatment, although ANP treatment also decreased the lipid droplet size in NFD mice as well (Fig. 3a,c). In addition, HFD feeding induced crown-like structures in epididymal WAT (eWAT), indicating tissue inflammation^22^, which was again attenuated by ANP treatment (Fig. 3a,d). Likewise, ANP treatment dramatically attenuated HFD-induced hepatic steatosis (Fig. 3a,e). In detail, HFD feeding led to the development of macrovesicular steatosis, lobular inflammation and hepatocellular ballooning, all of which were dramatically attenuated by exogenous ANP treatment. Accordingly, the non-alcoholic fatty liver disease (NAFLD) score as well as the steatosis score were significantly higher in HFD mice in comparison to NFD mice, both of which were decreased in ANP-treated HFD mice (Fig. 3e). These reversible effects of ANP on various adipose tissues and the liver may contribute to the improvement in HFD-induced insulin resistance.

Serum triglyceride^23^ and free fatty acid levels^18^ during the fasting state were comparable between NFD and HFD, consistent with the previous studies (Supplementary Table). Moreover, ANP treatment did not significantly affect them in a manner that was consistent with the previous study^18^. On the other hand, we found that the hepatic triglyceride content was increased in HFD, which is a typical finding of NAFLD^24^, and this was significantly decreased by ANP-treatment (Fig. 3e), reflecting the finding that ANP treatment ameliorated hepatic steatosis.

To study the effects of ANP on hepatic gluconeogenesis, the protein expression levels of key gluconeogenic enzymes in the hepatic tissues were evaluated (Supplementary Fig. S2). We found that phosphoenolpyruvate carboxykinase (PEPCK) levels were decreased in HFD, while glucose-6-phosphate (G6P) levels were comparable between groups. It can be stated that—at least—the ANP-treatment did not significantly affect the protein expression levels of both enzymes; however, it cannot be denied that ANP has a significant impact on the activities of these enzymes. Further studies, such as metabolome analyses^25^, are warranted to fully delineate the role of NPs in hepatic gluconeogenesis regulation.

HFD could also induce renal dysfunction due to the increased renal tissue fat volume, and exogenous ANP treatment could ameliorate it, given the renoprotective properties of NPs^26^. We therefore measured serum creatinine levels and found that they were comparable between NFD and HFD, at least in the present model, and that ANP treatment did not significantly affect these levels (Supplementary Table).

### Effects of ANP treatment on UCP1 expression in adipose tissues

**Figure 4:**
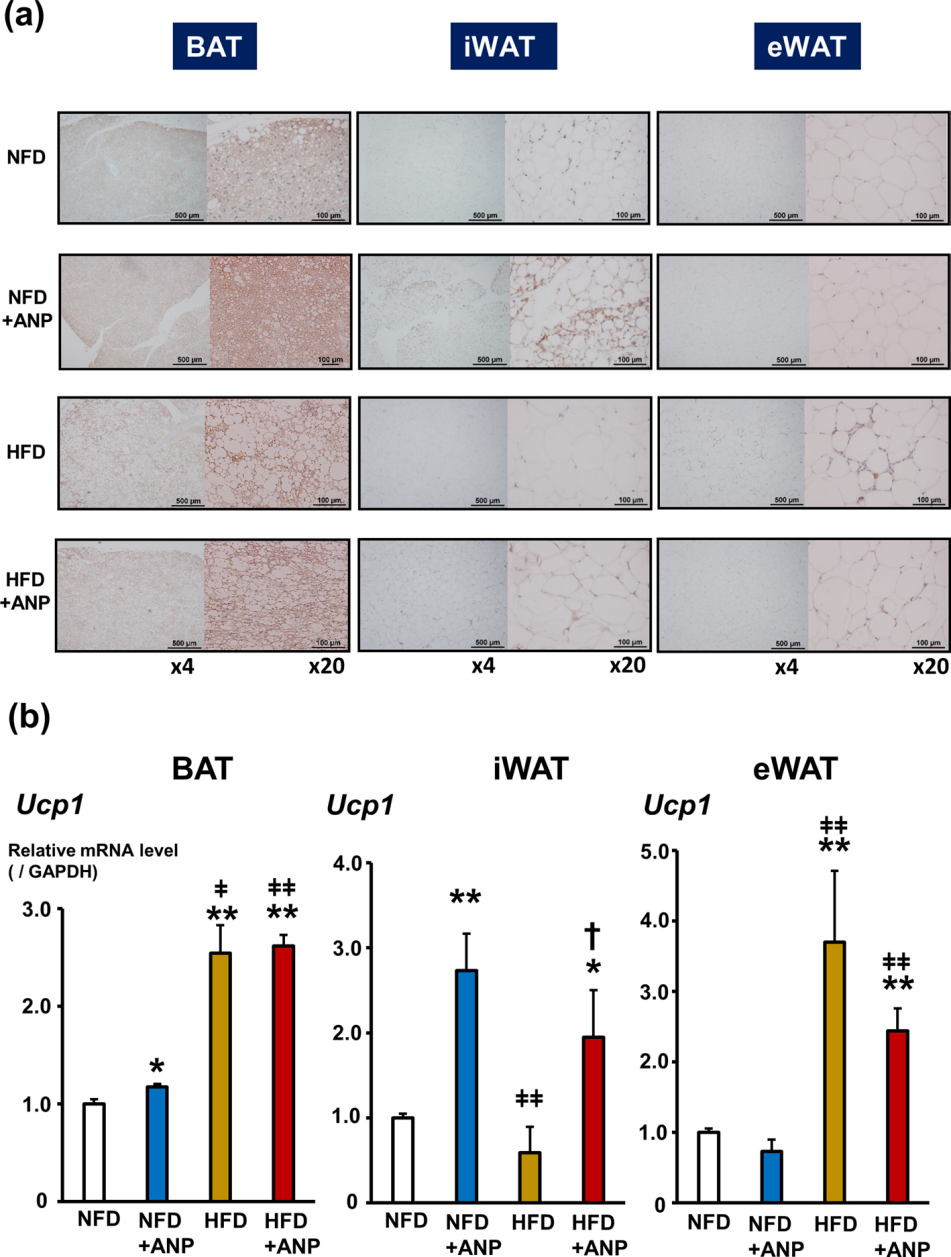
Effects of ANP treatment on UCP1 expression in adipose tissues. (**a**) Representative images of UCP1 immunostaining in each adipose tissue harvested from the indicated mice at three weeks after treatment with or without ANP (n = 3 each). The images were captured at a field lens magnification of × 4 (left, bars = 500 μm) and × 20 (right, bars = 100 μm). (**b**) The relative mRNA expression of UCP1 in each adipose tissue harvested from the indicated mice at three weeks after treatment with or without ANP (n = 5 each). The qPCR data were normalized to GAPDH. The data are shown as the fold change normalized to the levels found in NFD group. Data are mean ± SEM. *P < 0.05 and **P < 0.01 versus NFD; ^†^*P* < 0.05 versus HFD; ^ǂ^*P* < 0.05 and ^ǂǂ^P < 0.01 versus NFD + ANP. GAPDH, glyceraldehyde-3-phosphate dehydrogenase; UCP1, uncoupling protein 1.

UCP1, which is specifically expressed in BAT, plays an important role in the improvement of glucose tolerance and insulin sensitivity as well as thermogenesis^9^. Thus, we next examined the UCP1 expression in various adipose tissues in the current experimental model (Fig. 4 and Supplementary Fig. S3). Histological analyses (Fig. 4a) and biochemical analyses of mRNA (Fig. 4b) revealed that ANP treatment markedly increased the UCP1 expression in iWAT in both NFD and HFD mice, i.e. “*browning*” of WAT, although—for reasons which remain unknown—there was a problem with protein extraction from iWAT for immunoblotting. ANP treatment also increased the UCP1 expression in BAT in NFD mice, presumably via the activation of p38 mitogen-activated protein kinase (MAPK) pathway (Supplementary Fig. S3a and S3b)^6,9^, but not in HFD mice. Intriguingly, HFD per se increased the UCP1 expression in both BAT and eWAT, which was not affected by ANP treatment. These data suggest that each adipose tissue has a distinct role in metabolic regulation in response to ANP treatment and also the dietary condition.

### In vivo heat-retaining property of ANP during cold exposure

**Figure 5:**
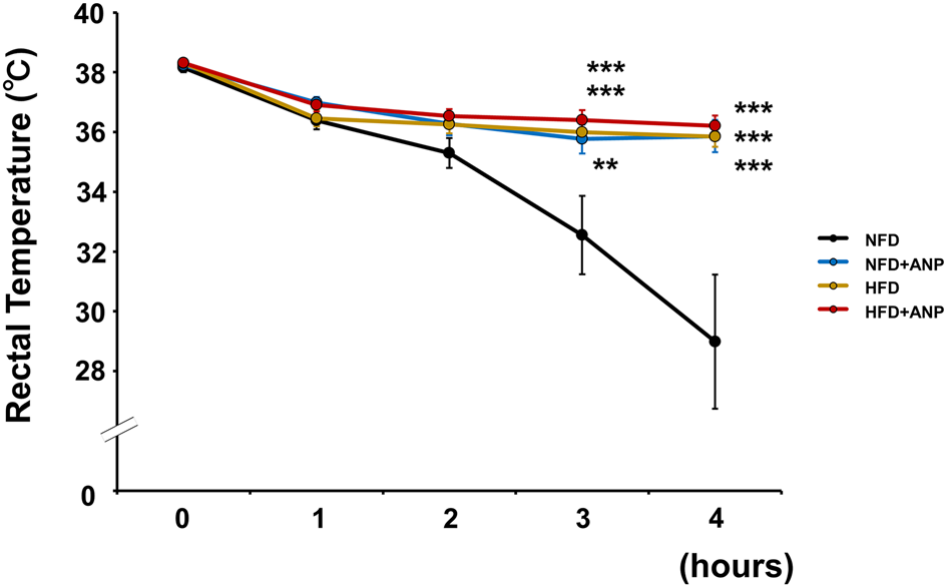
Changes in rectal temperature during acute cold exposure. Rectal temperature profiles during cold exposure at 4 °C for 4 h in the indicated mice at 3 weeks after treatment with or without ANP (n = 10 each). Data are mean ± SEM. **P < 0.01 and ***P < 0.001 versus NFD at each time point.

To test the functional significance of ANP-induced activation of the thermogenic program in adipose tissues, we investigated body temperature in cold-exposed mice with or without ANP treatment (Fig. 5). The rectal temperature in NFD mice gradually decreased over time during cold exposure at 4 °C for 4 h. In contrast, NFD mice with ANP treatment maintained euthermia upon cold exposure. HFD mice with and without ANP treatment were also tolerant to cold exposure (decrease in rectal temperature from baseline after 4-h cold exposure [°C]: NFD + ANP, − 2.4 ± 0.6 °C; HFD, − 2.5 ± 0.4 °C; HFD + ANP, − 2.1 ± 0.4 °C; NFD, − 9.2 ± 2.2 °C; *P* < 0.01 each, n = 10 each).

Finally, the serum ANP and BNP levels were measured at room temperature or at the end of the four-hour cold tolerance test (Supplementary Fig. S4). We found that the serum ANP levels as well as BNP levels were increased after cold exposure in both NFD and HFD mice, which was consistent with the findings of previous studies^6,7^, although the differences in BNP levels in HFD mice failed to reach statistical significance (*P* = 0.099).

## Discussion

In the present study, we found that exogenous ANP administration significantly improved the HFD-induced insulin resistance by attenuating hepatic steatosis and inducing adipose tissue browning in association with the activation of the brown fat thermogenic program. Accordingly, the mice treated with ANP developed tolerance to cold exposure, indicating the adaptive heat-retaining property of NPs when body temperature falls via interorgan metabolic crosstalk with adipose tissues. The remarkable findings in the present study are that the systemic administration of exogenous ANP has a substantial impact on the morphology and the features of the various adipose tissues and hepatic tissue, leading to the reduction in systemic insulin resistance induced by HFD, and most notably, in vivo thermogenesis during cold exposure.

BAT, as opposed to WAT, mainly functions to dissipate energy and promote heat production using metabolic fuel, thus causing it to be considered the major site of non-shivering thermogenesis^7,13,14,27^. BAT was originally thought to have evolved as a compensatory defense system against hypothermia in mammals^9,28^. By increasing energy expenditure, the activation of BAT also functions to improve whole-body glucose homeostasis and plays a critical role in mitigating the deleterious effects of obesity and diabetes^29–31^. Accumulating evidence suggests that NPs not only stimulate lipolysis by binding to the receptor NPR-A and subsequently activating the cGMP-PKG pathway but also activate BAT via the further upregulation of UCP1^9,14,19^. More interesting is the fact that NPs induce the functional phenotype characteristics of brown adipocytes in WAT, so-called browning of fat, which results in an increase in the thermogenic energy expenditure capacity^19^. In fact, the present study clearly showed that ANP treatment reconstitutes and reorganizes the morphological and functional features of both BAT and WAT in vivo, particularly in the setting of HFD-induced obesity. Specifically, ANP treatment promotes the recruitment of brown-like (beige) or brown-in-white (brite) adipocytes to iWAT with enlarged lipid droplets as well as to BAT with *whitening* under HFD conditions, as evidenced by the increased emergence of adipocytes containing multilocular lipid droplets and enhanced UCP1 immunohistochemical staining^32^. Further studies on the expression levels of the markers of BAT activity, such as Dio2 and PGC1α, are warranted to fully delineate the precise pathway and mechanism underlying the activation of the thermogenic program by ANP treatment.

The present study indicates that BAT and WATs (iWAT and eWAT) play distinct roles in maintaining systemic energy homeostasis in response to either ANP treatment or HFD-induced obesity, as shown by the UCP1 expression in each adipose tissue. UCP1 is recognized as a molecular marker as well as an indicator of BAT activity and plays a key role in adaptive thermogenesis. ANP treatment induced UCP1 expression predominantly in iWAT, regardless of the diet, although it also increased UCP1 in BAT under NFD conditions. In contrast, HFD per se induced UCP1 expression predominantly in BAT and eWAT, which is consistent with the findings of previous studies^33–35^. The proposed mechanism is as follows: the elevation of circulating levels of various substrates under HFD condition, such as glucose and fatty acids, promotes the uptake of these substrates into BAT and eWAT. The increased supply of these substrates (as fuel) to those adipose tissues induces the expression and activation of UCP1 for adaptive thermogenesis^36,37^. Therefore, although HFD morphologically induced “lipid-rich BAT”, namely, BAT *whitening*, the UCP1 expression was rather increased reflecting the consumption of the augmented lipid droplets as an adaptive mechanism.

The mechanism by which ANP treatment ameliorated HFD-induced insulin resistance may involve morphological changes in each adipose tissue: namely, *re-browning* of lipid-rich BAT, browning of iWAT, and a reduction in the crown-like structure (indicating tissue inflammation) in eWAT. Furthermore, the augmented UCP1 expression by ANP treatment in iWAT might also play a role. Likewise, the findings that ANP treatment attenuated HFD-induced hepatic steatosis are in line with those of previous studies^18,20^, which also substantially contributes to ameliorating insulin resistance in HFD mice. Serum free fatty acid levels are generally elevated as a consequence of enhanced lipolysis. However, we found that serum triglyceride and free fatty acid levels were comparable between NFD and HFD—at least in the present model—and ANP treatment did not significantly affect them, which was consistent with the previous study^18^. This might be because the blood samples were collected after a substantial period of fasting. It is also possible that the effect of NPs on the lipid profiles in the circulating blood per se is not so prominent at baseline, particularly in experimental animal models, although the substantial impact of NPs on the metabolic profile becomes salient during some sort of external stimuli or intervention, such as glucose tolerance test or severe cardiac conditions.

The mechanism by which ANP treatment attenuated HFD-induced hepatic steatosis is of great interest. Although phospho-p38 levels were increased in HFD per se, consistent with the previous study^23^, ANP-treatment did not significantly affect them (Supplementary Fig. S3c). Thus, unlike adipose tissues, p38MAPK signal may not play a critical role in the attenuation of diet-induced hepatic steatosis by ANP-treatment. Given that oxidative stress has a central role in the pathogenesis of NAFLD^38,39^, it is also very likely that the anti-oxidative effects of ANP^40^ lead to the attenuation of hepatic steatosis. Further mechanistic studies including metabolome analyses^25^ are clearly warranted to fully delineate the effects of ANP treatment on the fatty acid oxidation pathways, as well as hepatic gluconeogenesis regulation in models of diet-induced obesity.

One can speculate that ANP does not have a marked impact on obesity per se based on the current finding of a lack of body weight change in ANP-treated HFD mice, in a manner that was consistent with the previous study^41^. In fact, the previous studies showed that the downregulation of NPR-A expression and upregulation of NP degradation systems (i.e., NP clearance receptor [NPR-C] and neprilysin) are observed in obese or diabetic subjects^27,41,42^, which may result in the blunted impact of NPs on obesity. However, other studies showed that prolonged activation of NPs cascades reduced body weight gain in obese subjects^18,20,43^; thus, it is highly likely that exogenous ANP at a higher dose for a longer treatment period could have an anti-obesity effect. In any case, the present study clearly showed that exogenous ANP—even at a dose that does not affect either blood pressure or body weight—may still have a significant influence on the “metabolic tissues”, such as adipose tissues and the liver, leading to the improvement in HFD-induced insulin resistance, the cornerstone of the pathophysiology of obesity. In other words, it turns out that not only cardiovascular and renal tissues but also adipose and hepatic tissues are highly sensitive to NPs. Furthermore, the exogenous administration of ANP ameliorates HFD-induced insulin and glucose tolerance through inter-organ metabolic crosstalk, even without any changes in body weight, namely by attenuating hepatic steatosis and inducing adipose tissue browning (activation of the adipose tissue thermogenic program).

The mechanism by which the mice treated with ANP developed tolerance to cold exposure is proposed to involve exogenous ANP activating the thermogenic program in BAT and iWAT. Consistent with the present findings, our previous in vitro study showed that ANP increased the UCP1 expression via the p38MAPK pathway in brown adipocytes^9^. However, a recent study indicated an essential role of WAT lipolysis and browning as a cold adaptive mechanism, especially during fasting^32,37^. In fact, the present study showed that augmented WAT browning by ANP treatment observed in iWAT, as evidenced by the increased emergence of adipocytes containing multinodular lipid droplets and enhanced UCP1 expression, is more salient than that in other adipose tissues, suggesting that iWAT plays a central role in developing cold tolerance in the present experimental model.

The mice fed HFD alone also developed cold tolerance presumably through UCP1 upregulation in BAT and eWAT, as noted above. Why ANP treatment did not exert further thermogenic action in HFD mice may involve the following: NPs exert warming effects in a low-temperature-sensitive manner, as also indicated by our previous experimental^9^ and clinical^4^ studies. Therefore, NPs might have minimal effects on the temperature regulation under relatively high temperature conditions (i.e. thermoneutral conditions), as in the case of an HFD, which induces cold tolerance by itself. Other possible mechanisms are upregulation of the degradation systems of NPs (i.e. NPR-C and neprilysin) and/or an impaired adipose tissue function in HFD-induced obesity. However, neither of them might be the major mechanism, given that exogenous ANP showed a significant influence on the morphology of the adipose and hepatic tissues in HFD mice, consequently substantially ameliorating the HFD-induced insulin resistance.

We found that circulating NPs levels are elevated during cold exposure, which is consistent with the findings of previous studies, including our own^6,7,10^. Several possible mechanisms have been proposed. One is that, in response to cold, superficial blood vessels contract in order to limit heat loss, causing blood to be shunted away toward deeper large blood vessels that increase the cardiac filling pressure, thereby increasing the NP production/secretion in the heart^7^. Alternatively, the NPR-C expression in adipose tissues is decreased during cold exposure^6,7^, so NP degradation is reduced. Although the precise mechanisms remain to be elucidated, these findings suggest the existence of an adaptive biological response to a cold environment via myocardial-adipose crosstalk.

Several limitations associated with the present study warrant mention. First, it was reported that the expression of NPR-C in rodents is approximately 100-fold higher than that in humans^6,9^. However, previous studies have shown that during fasting conditions, the expression of NPR-A was upregulated, while the expression of NPR-C was downregulated^18,44^. In the present study, the experiments were performed basically under fasting conditions. In addition, exogenous ANP was administered at a pharmacological dose, although it did not affect either the blood pressure profile or body weight. Taken together, our findings suggest that exogenous ANP may still have a significant influence, even in the rodent model used in the present study. Further investigation on the tissue cGMP levels is required to assess target engagement^45^. Second, the results might be different if body temperature is measured by an implantable telemetry device^32,37,46,47^, particularly when considering that the body temperature response is compared between lean and obesity subjects. One can speculate that rectal temperature would not be an ideal readout given that this procedure is stressful for animals and that only a limited number of data points can be collected, although several previous studies also used the rectal temperature to monitor core body temperature during cold exposure^7,30,34,48^. Lastly, we found that circulating NP levels were increased under HFD conditions, which was consistent with the previous findings^43^. Various clinical studies have indicated that obese patients with or without heart failure show unexpectedly low NP levels^5,49,50^. The reason for the discrepancy between the clinical results and these experimental findings remains to be determined. However, research investigating the impact of exogenous ANP on the heart, such as the cardiac function, myocardial morphology, and myocardial adipose tissue, in the present model of diet-induced obesity is underway in our laboratory.

In conclusion, the systemic administration of ANP has a substantial impact on the morphology and features of the various adipose tissues and hepatic tissue. Exogenous ANP ameliorates HFD-induced insulin resistance by promoting adipose tissue browning as well as by attenuating hepatic steatosis. Notably, ANP treatment induces cold tolerance by activating the adipose tissue thermogenic program in vivo. The present study uncovered a previously underappreciated role of NPs in energy metabolism regulation (the derangement of which is a hallmark of the pathogenesis of heart failure^51,52^) through inter-organ metabolic crosstalk. Given that insulin resistance is highly prevalent in the heart failure population^51,52^ and that a low body temperature is associated with a worse outcome in patients with worsening heart failure^53,54^, the administration of agents that increase circulatory NP levels may have therapeutic benefits from the perspective of the present study.

## Methods

### Animal models

All animal procedures conformed to the National Institutes of Health Guide for the Care and Use of Laboratory Animals and were approved by the Animal Research Committee at the Jikei University School of Medicine (2016-038C8). All animal experiments were carried out in accordance with the ARRIVE guidelines. The study design is shown in Fig. 1a. Male C57BL/6 mice at 8 weeks of age were fed either NFD or HFD for 13 weeks as previously described^55^. Where indicated, mice fed an NFD or HFD received ANP (carperitide, kindly provided by Daiichi-Sankyo Pharmaceutical Co.) subcutaneously via a mini-osmotic pump (model 2004; Alzet Corporation, CA, USA) for three weeks. ANP was dissolved in sterile water to a final concentration of 6 mg/ml. The mini-osmotic pumps were filled with 250 μl of the ANP solution and set to release 0.25 μl/h (0.5 μg/kg/min). The control group received pumps containing sterile water only. All mice were housed at room temperature (25 °C). Body weight and blood pressure were measured weekly during the study period. The blood pressure in conscious mice was measured using a noninvasive computerized tail-cuff system (BP-98A-L, Softron Co., Ltd., Tokyo, Japan). Mice were held in a small mouse pocket on a warming pad thermostatically controlled at 37 °C. Systolic and diastolic pressure were calculated at three different times and averaged.

At the end of the experimental period (Fig. 1a), the mice were euthanized (60 mg/kg of pentobarbital, intraperitoneally [i.p.]) in order to eliminate suffering. The liver, eWAT, iWAT, and BAT were then dissected from mice and washed in phosphate-buffered saline (PBS) (FUJIFILM, Wako Pure Chemical Corporation, Japan) at 4 °C. After washing, tissues were flash-frozen in liquid nitrogen and stored at − 80 °C until further analyses. Some portions of the adipose tissues and liver were isolated for histological studies.

### Glucose and insulin tolerance tests

Three weeks after the treatment with or without ANP, we performed IPGTT and ITT as previously described^55^.

### Cold tolerance test

Three weeks after treatment with or without ANP, mice were fasted for 16 h, and at 7 a.m., they were individually housed at 4 °C for 4 h with free access to water. Rectal temperature was measured every hour during cold exposure using an MC1000 bio-research thermometer (Tokai Hit, Shizuoka, Japan). At the end of the study period, blood was collected for the serum ANP and BNP measurement, as described below.

### Triglyceride, Free Fatty Acid, creatinine, and insulin measurements in serum

The blood was drawn from the indicated mice and put on ice. After centrifuged (3000 g, 15 min, 4 °C), the serum (supernatant) was collected into new tubes and frozen at − 80 °C prior to the measurement. The concentrations of triglyceride, free fatty acid, and creatinine were measured and analyzed using LabAssay Triglyceride (#LABTR G-M1, Fujifilm Wako Pure Chemical Corporation, Tokyo, Japan), LabAssay NEFA (#294-63601, Fujifilm Wako Pure Chemical Corporation, Tokyo, Japan), and LabAssay Creatinine (#290-65901, Fujifilm Wako Pure Chemical Corporation, Tokyo, Japan), respectively, according to the manufacture’s protocols. The measurement of serum insulin concentration was conducted by SRL, Inc. (Tokyo, Japan).

### ANP and BNP measurement in serum

The blood was drawn from the indicated mice housed at room temperature or after cold exposure for 4 h. The blood was collected into micro tubes containing aprotinin (#9087-70-1; Wako, Tokyo, Japan), a protease inhibitor, and placed on ice. Each sample was centrifuged (3000 r.p.m., 15 min, 4 °C) and the serum (supernatant) was stored at − 80 °C. Serum ANP and BNP levels were measured using RayBio Mouse ANP Enzyme Immunoassay Kit (#EIA-ANP; RayBiotech, GA, USA) and BNP Enzyme Immunoassay Kit (#EIA-BNP; RayBiotech), respectively.

### Histological analyses

Liver, eWAT, iWAT and BAT were excised, washed in ice-cold PBS, and fixed with 10% formalin. The samples were embedded into paraffin, and 8 µm sections were prepared for following histological analyses as previously described^56^. Hematoxylin and eosin-stained sections were visualized using ECLIPS 80i (Nikon Co., Tokyo, Japan). The images were captured at a field lens magnification of × 4 (left, bars = 500 μm) and × 20 (right, bars = 100 μm). The cell size of iWAT (total of 150 cells per field in each group) was analyzed and the size of lipid droplets in BAT and liver were measured using ImageJ Software at a field lens of × 20. The number of crown like structures in eWAT was calculated at a field lens magnification of × 4. Large lipid droplets in BAT and liver were defined as lipids larger than 100 μm^2^, measured with ImageJ, and counted per field at a field lens of × 20 magnification, according to the previous study with some modifications^46^. We evaluated liver histology and grading liver injury using a scoring system for nonalcoholic steatohepatitis (NASH) established by Savari et al.^57^. According to this standardized scoring system, the sum of steatosis (0–3), lobular inflammation (0–3), and hepatocellular ballooning degeneration (0–2) scores considered to be the NAFLD activity score. The criteria were as follows: steatosis grade, 0: less than 5%, 1: between 5 and 33%, 2: between 33 and 66%, and 3: more than 66%. For lobular inflammation, minimal or absence of inflammatory cells accumulation (infiltration) scored as grade 0, mild infiltration (grade 1), moderate to severe infiltration (grade 2) and severe inflammatory cells 8 accumulation (grade 3). Cell ballooning scored as none (0), mild (few swelled cells; 1) and severe (many swelled cells; 2) based on its severity.

For immunohistochemical staining, fixed adipose tissue sections were incubated with rabbit polyclonal anti-UCP1 antibody (1:500) (U6382; Sigma-Aldrich, Tokyo, Japan). The stained images were visualized and captured using ECLIPS 80i.

### Tissue triglyceride content in the liver

The hepatic tissue triglyceride content was measured using triglyceride quantification kit (Cell Biolabs Inc. # STA-396, United States). Frozen samples of liver (100 mg) were homogenized in 1 mL of cold PBS containing 1% TritonX-100 and centrifuged (10000 g, 10 min, 4 °C). The resulting supernatant extracted from the tissue homogenates were carefully collected and further diluted in the ratio 1:5. The resultant lysates were analyzed according to the manufacture’s protocols, and the tissue triglyceride content was corrected based on the tissue weight.

### Immunoblotting

Immunoblotting was performed as previously described^9,25,55^ with mouse monoclonal anti-PEPCK (1:10,000, sc-271029; Santa Cruz, United States), rabbit monoclonal anti-G6P (1:1000, #12263U; Cell Signaling Technology, Tokyo, Japan), rabbit polyclonal anti-UCP1 (1:5000, U6382; Sigma-Aldrich, Tokyo, Japan), rabbit monoclonal anti-phospho-p38 (1:1000 (BAT), 1:5000 (liver), # 4511; Cell Signaling Technology, Tokyo, Japan), rabbit monoclonal anti-total p-38 (1:3000, # 8690; Cell Signaling Technology, Tokyo, Japan), and rabbit monoclonal anti-GAPDH (1:5000, #2118; Cell Signaling Technology, Tokyo, Japan). The signals were detected using chemiluminescence.

### RNA isolation, reverse transcription (RT) and real-time polymerase chain reaction (PCR)

Total RNA was extracted from the frozen tissues using TRIzol reagent (Invitrogen) and a quantitative real-time PCR was performed using a StepOnePlus Real-time PCR System and the StepOne Software program (Applied Biosystems), as described previously^9,55^. The real-time PCR protocol consisted of one cycle at 95 °C for 20 s followed by 40 cycles at 95 °C for 1 s and 60 °C for 20 s using the primers for UCP1 (Mm01244861_m1; Applied Biosystems). The transcriptional levels were determined using the ΔΔCt method with normalization to GAPDH (Mm99999915_g1; Applied Biosystems).

### Statistical analyses

The data are presented as the mean ± standard error of the mean (SEM) of at least three independent experiments. For the comparison of two datasets, Student’s *t*-test was performed. For multiple comparisons among ≥ 3 groups, one-way analysis of variance (AONVA) with Bonferroni’s method for post hoc comparisons and non-parametric Kruskal–Wallis test and Mann–Whitney *U* test were performed. A value of *P* < 0.05 was considered to be significant.

## Supplementary Information

Supplementary Information.

## Data Availability

The datasets generated during and/or analysed during the current study are available from the corresponding author on reasonable request.
