# Supplementary material for: Treatment with atrial natriuretic peptide induces adipose tissue browning and exerts thermogenic actions in vivo
Source: Sci Rep. 2021 Aug 31;11:17466. doi: 10.1038/s41598-021-96970-9 (PMC8408225; doi:10.1038/s41598-021-96970-9)
Supplement: Supplementary file 1 — Supplementary Information. [file 41598_2021_96970_MOESM1_ESM.docx]

**Supplementary Information**

**Treatment with atrial natriuretic peptide induces adipose tissue browning and exerts thermogenic actions *in vivo*.**

Haruka Kimura^¶1^, Tomohisa Nagoshi^¶^*^1^, Yuhei Oi^1^, Akira Yoshii^1^, Yoshiro Tanaka^1^, Hirotake Takahashi^1^, Yusuke Kashiwagi^1^, Toshikazu D. Tanaka^1^, and Michihiro Yoshimura^1^

[^¶^These authors contributed equally to this work.]

^1^ Division of Cardiology, Department of Internal Medicine,

The Jikei University School of Medicine

***Correspondence to:** Tomohisa Nagoshi, M.D.,Ph.D.

E-mail: [tnagoshi@jikei.ac.jp](mailto:tnagoshi@jikei.ac.jp)

**Supplementary Table. Blood serum sample analyses**

|  | NFD | NFD+ANP | HFD | HFD+ANP |
| --- | --- | --- | --- | --- |
| Triglyceride (mg/dl) | 44.2±1.70 | 40.8±7.99 | 43.13±3.58 | 50.21±6.99 |
| Free fatty acid (mEq/l) | 0.89±0.12 | 0.79±0.12 | 0.73±0.10 | 0.71±0.10 |
| Creatinine (mg/dl) | 0.66±0.08 | 0.62±0.06 | 0.61±0.03 | 0.62±0.05 |

Triglyceride and Free fatty acid; n=6, Creatinine; n=3

**
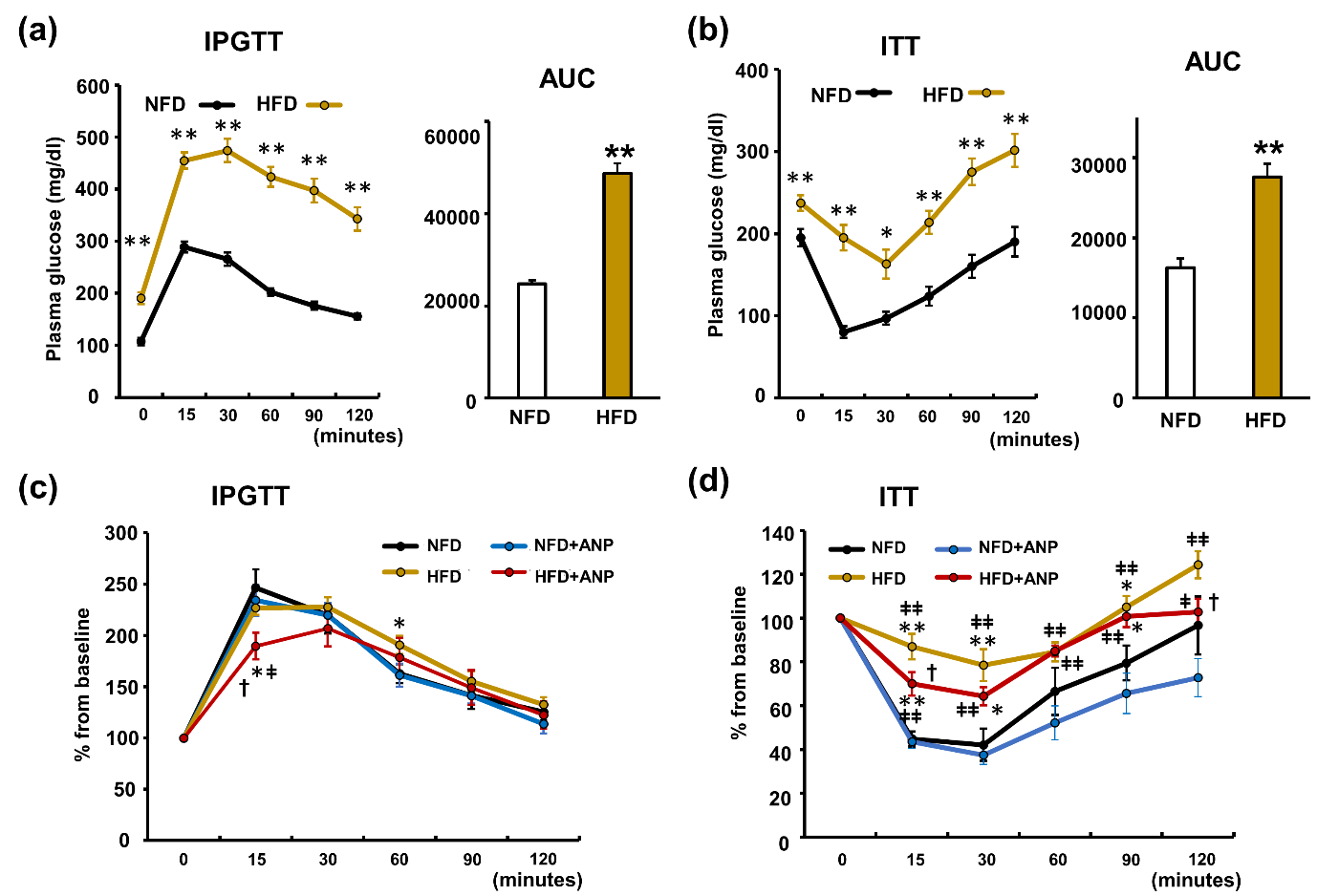
**

**Fig. S1 Thirteen-week HFD feeding induced insulin resistance and ANP treatment ameliorated it.** Plasma glucose levels during IPGTT **(a)** and ITT **(b)** before ANP treatment (n=14 each). The area under the curve (AUC) was calculated from the plasma glucose levels profile shown in each test. Plasma glucose levels (% from baseline) during IPGTT **(c)** and ITT **(d)** at three weeks after treatment with or without ANP ((c) NFD and HFD, n=8 each; NFD+ANP, n=9; HFD+ANP, n=7. (d) NFD, NFD+ANP and HFD, n=9; HFD+ANP, n=8.). Data are mean ± SEM. *P<0.05 and **P<0.01 vs. NFD; ^†^P<0.05 and ^††^P<0.01 vs. HFD; ^ǂ^P<0.05 and ^ǂǂ^P<0.01 vs. NFD+ANP at each time point. ANP, A-type natriuretic peptide; HFD, high-fat diet; IPGTT, intraperitoneal glucose tolerance test; ITT, insulin tolerance test; NFD, normal-fat diet.

**
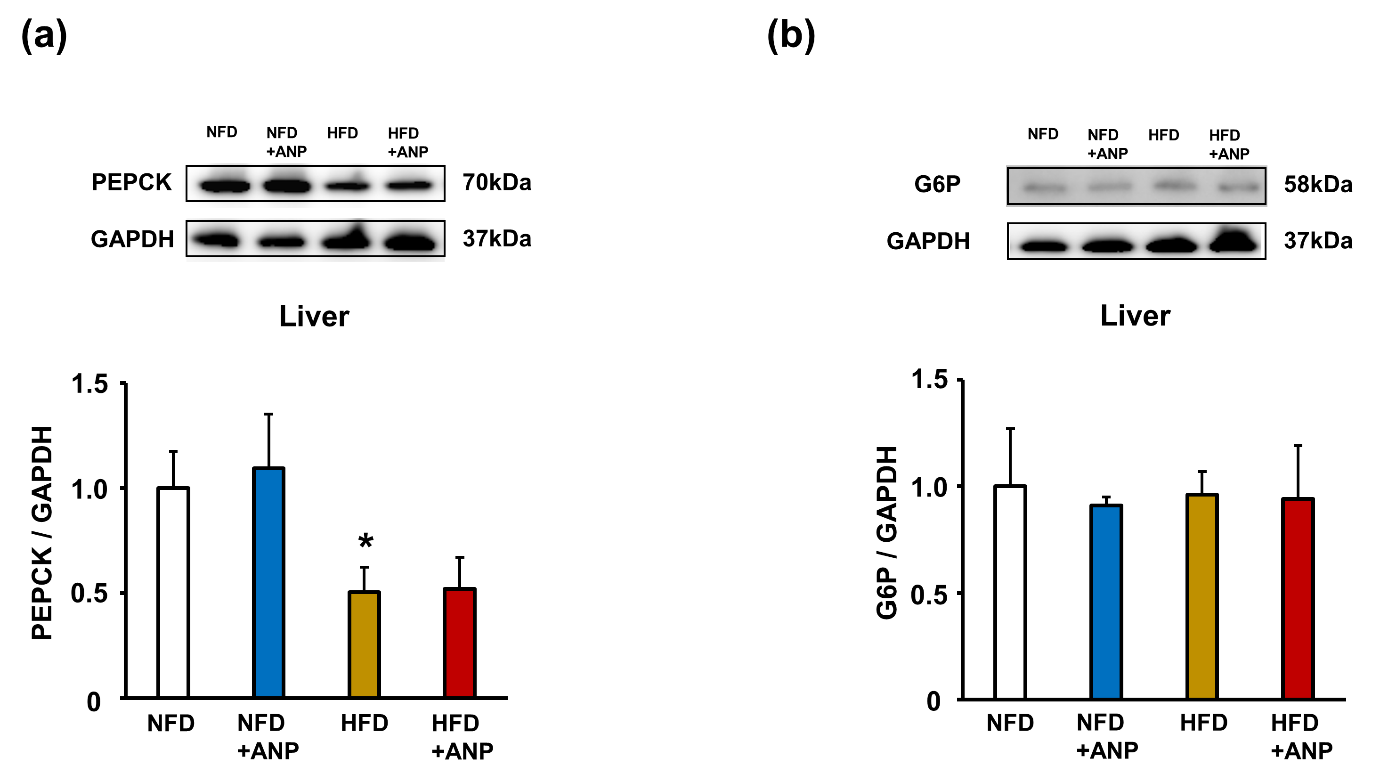
**

**Fig. S2 The protein expression levels of hepatic gluconeogenic enzymes in NFD and HFD mice treated with or without ANP.** Representative immunoblots obtained using the indicated antibodies (PEPCK **(a)** and G6P **(b)**) are shown and full-length blots are presented in Supplementary Fig. S5a. Averaged densitometry data normalized to the NFD subjects are shown in the bar graphs (PEPCK, n=6 each; G6P, n=3 each). Data are mean ± SEM. *P<0.05 vs. NFD. PEPCK, phosphoenolpyruvate carboxykinase; G6P, Glucose-6-phosphate; GAPDH, glyceraldehyde-3-phosphate dehydrogenase.

**
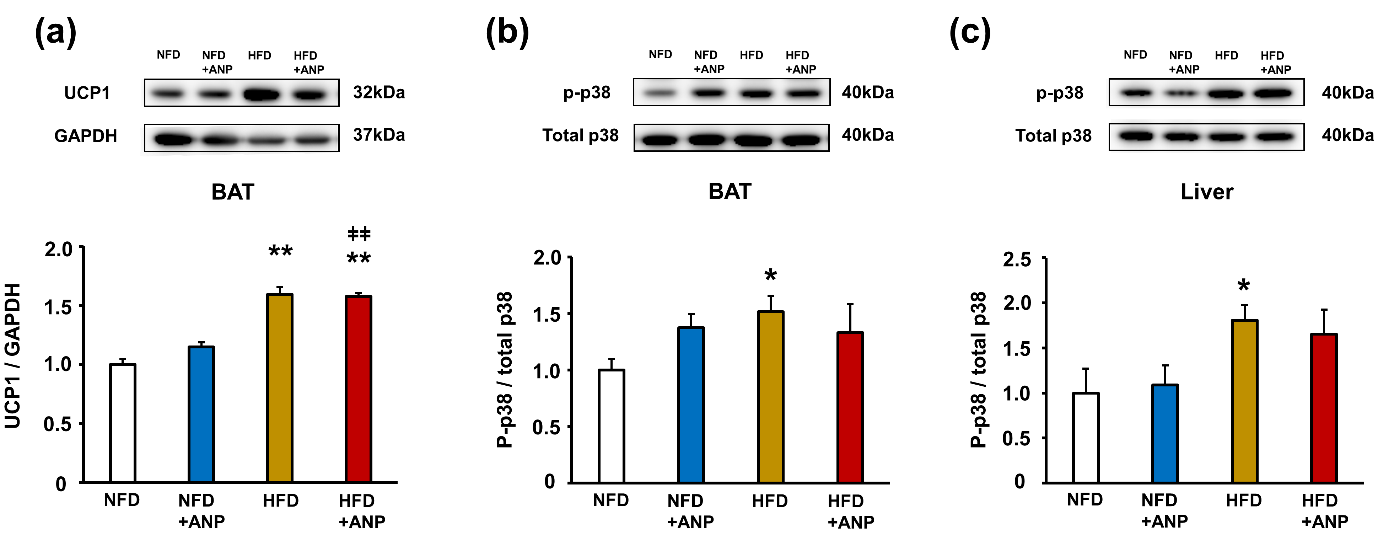
**

**Fig. S3 The effects of ANP on UCP1 expression and phosphorylation of p38MAPK in BAT and liver.** Representative immunoblots obtained using the indicated antibodies are shown full-length blots are presented in Supplementary Figs. S5b and c. Averaged densitometry data normalized to the NFD subjects are shown in the bar graphs (UCP1 **(a)** and phospho- / total p38 **(b)** in BAT, n=3 each; phospho- / total p38 in liver **(c)**, n=6 each). Data are mean ± SEM. *P<0.05 and **P<0.01 vs. NFD; ^ǂǂǂǂ^P<0.01 vs. NFD+ANP. UCP1, uncoupling protein 1; p-p38, phospho-p38.

**
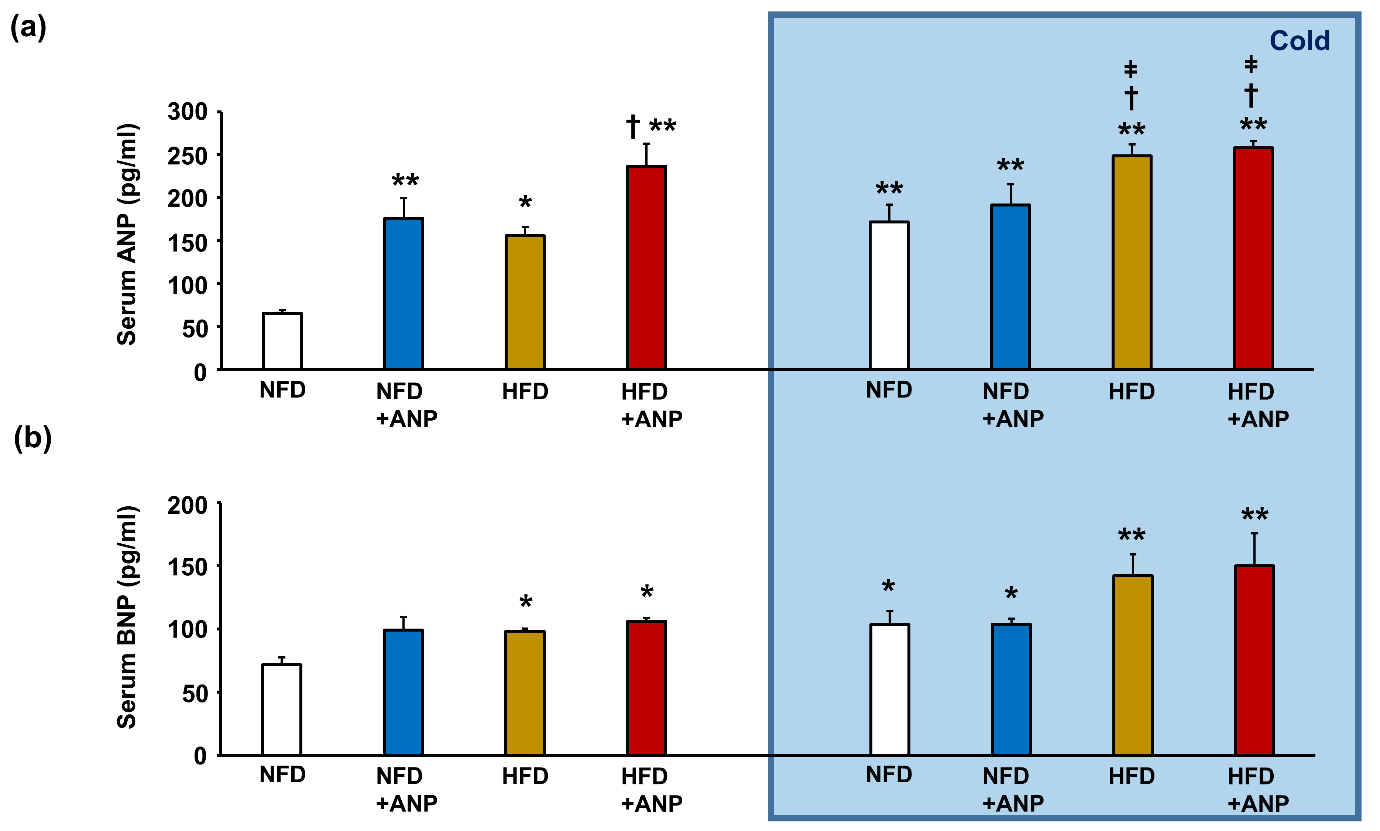
**

**Fig. S4 Serum levels of natriuretic peptides in NFD or HFD mice at room temperature and after acute cold exposure.** The serum concentrations of ANP **(a)** and BNP **(b)** in the indicated mice at 3 weeks after the treatment with or without ANP are shown (room temperature [25°C], n=3 each; cold exposure [4°C], n=5 each). Data are mean ± SEM. *P<0.05 and **P<0.01 vs. NFD; ^†^P<0.05 vs. HFD; ^ǂ^P<0.05 vs. NFD after cold exposure. BNP, B-type natriuretic peptide.


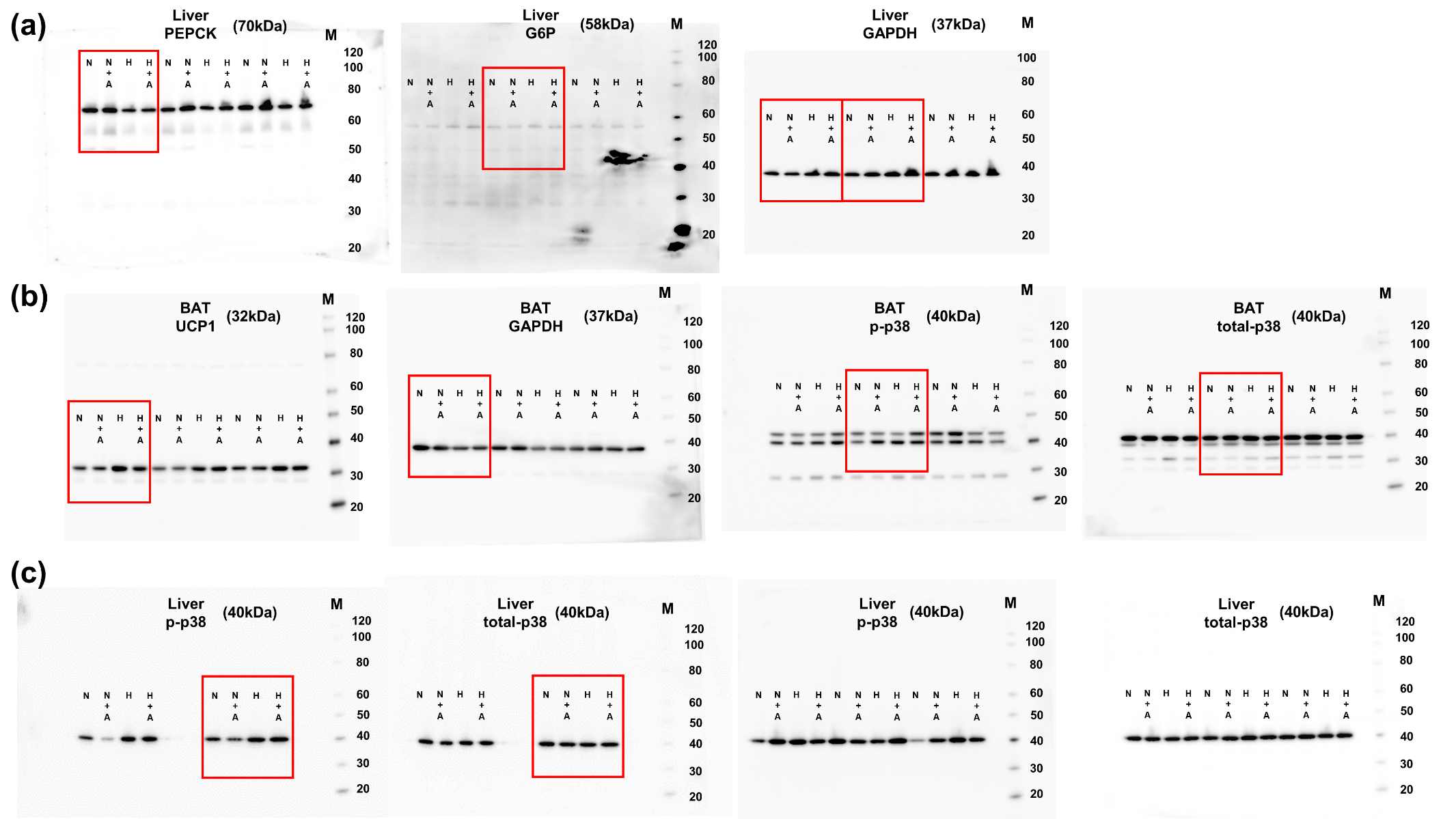


**Fig. S5 Full-length gels of immunoblottings.**

Western blotting of the indicated antibodies. Red boxes correspond to the cropped samples shown in Suppl. Fig. S2 and S3. A, ANP; H, high-fat diet; kDa, kilo Dalton; M, MagicMark XP Western Protein standard, a molecular weight marker; N, normal-fat diet.
